# Preterm birth: Case definition & guidelines for data collection, analysis, and presentation of immunisation safety data

**DOI:** 10.1016/j.vaccine.2016.03.045

**Published:** 2016-12-01

**Authors:** Julie-Anne Quinn, Flor M. Munoz, Bernard Gonik, Lourdes Frau, Clare Cutland, Tamala Mallett-Moore, Aimee Kissou, Frederick Wittke, Manoj Das, Tony Nunes, Savia Pye, Wendy Watson, Ana-Maria Alguacil Ramos, Jose F. Cordero, Wan-Ting Huang, Sonali Kochhar, Jim Buttery, The Brighton Collaboration Preterm Birth Working Group

**Affiliations:** a SAEFVIC, Murdoch Childrens Research Institute, Victoria, Australia; b Infection and Immunity, Monash Children's Hospital, Department of Paediatrics, The Ritchie Centre, Hudson Institute, Monash University, Australia; c Departments of Pediatrics and Molecular Virology and Microbiology, Baylor College of Medicine, Houston, TX, USA; d Department of Obstetrics and Gynecology, Wayne State University, Detroit, MI, USA; e LMF Pharmacoepidemiology, New York, USA; f Medical Research Council: Respiratory and Meningeal Pathogens Research Unit, Department of Science and Technology National Research Foundation, Vaccine Preventable Diseases, Faculty of Health Sciences, University of the Witwatersrand, Johannesburg, South Africa; g Global Pharmacovigilance, Sanofi Pasteur, Swiftwater, Philadelphia, USA; h Department of Pediatrics, Souro Sanou Teaching Hospital, Bobo-Dioulasso, Burkina Faso; i Debiopharm International SA, Lausanne, Switzerland; j INCLEN Trust International, India; k Optum Epidemiology, Providence, RI, USA; l Communicable Disease Prevention and Control, Nova Scotia, Canada; m Pfizer Inc, Collegeville, PA, USA; n Dirección General de Salud Pública, Avd Cataluña n°21, 46020 Valencia, Spain; o University of Puerto Rico Graduate School of Public Health, Medical Sciences Campus, San Juan 00935, Puerto Rico; p Taiwan Centers for Disease Control, Taipei, Taiwan; q Global Healthcare Consulting, India

**Keywords:** Preterm birth, Adverse event, Antenatal, Immunisation, Guidelines, Case definition

## Abstract

Preterm birth is commonly defined as any birth before 37 weeks completed weeks of gestation. An estimated 15 million infants are born preterm globally, disproportionately affecting low and middle income countries (LMIC). It contributes directly to estimated one million neonatal deaths annually and is a significant contributor to childhood morbidity. However, in many clinical settings, the information available to calculate completed weeks of gestation varies widely. Accurate dating of the last menstrual period (LMP), as well as access to clinical and ultrasonographic evaluation are important components of gestational age assessment antenatally. This case definition assign levels of confidence to categorisation of births as preterm, utilising assessment modalities which may be available across different settings. These are designed to enable systematic safety evaluation of vaccine clinical trials and post-implementation programmes of immunisations in pregnancy.

## 1 Preamble

### 1.1 Need for developing case definitions and guidelines for data collection, analysis, and presentation for preterm birth as an adverse event following immunisation

Preterm birth has been defined as any birth before 37 weeks completed weeks of gestation. An estimated 15 million infants are born preterm, with resulting complications. It is the principal cause of an estimated one million neonatal deaths annually and a significant contributor to childhood morbidities. Low and middle income countries (LMIC) carry a higher burden of disease attributed to preterm birth.

The World Health Organisation (WHO) defines preterm birth as any birth before 37 completed weeks of gestation, or fewer than 259 days since the first day of the woman's last menstrual period (LMP). This is further subdivided on the basis of gestational age (GA):

• extremely preterm (<28 weeks);
• very preterm (28–<32 weeks);
• moderate or late preterm (32–<37 completed weeks of gestation).

This is the most extensively used and accepted definition of preterm birth [1].

The ability to accurately determine the completed weeks of gestation varies widely between pregnancies, with the most precise assessment methods not uniformly available across different settings. Vaccination in pregnancy has been widely implemented to protect women and their babies from tetanus and pertussis in recent years, with an increasing number of vaccines being developed and trialled for use in pregnancy against a variety of bacterial and viral infections. As preterm birth is such an important pregnancy outcome that may represent an adverse event, it is important to establish a case definition for use across vaccine studies and post-licensure surveillance that is able to make use of all methodologies used to calculate gestational age, and that incorporates a hierarchy based upon the precision of the various methods used.

The nomenclature of GA is typically discussed in terms of the number of completed weeks (e.g., 33 weeks and 2 days, or 33 2/7 weeks). Defining GA has been considered useful in terms of neonatal outcome. In the past, three groups have been classified and utilised according to delivery following the onset of the last menstrual period. *Pre-term*: less than 259 days (37 weeks), *term*: 259–293 days (37–41 weeks). *Post-term*: 294 days (42 weeks) or more.

A term birth has been defined as between 37 and 42 weeks and used to describe the optimal timing for a good outcome for the mother and baby. The International Classification of Diseases defines term pregnancy as a delivery from 37 completed weeks to less than 42 completed weeks (259–293 days) of gestation. However, neonatal outcomes vary within this wide gestational age range, with a 2012 international stakeholder working group recommending sub-categorisation of term birth to more accurately describe deliveries and their outcomes. These sub-categories are: *early term* (37 0/7 weeks of gestation through 38 6/7 weeks gestation); *full term* (39 0/7 weeks of gestation through 40 6/7 weeks of gestation); *late term* (41 0/7 weeks of gestation through 41 6/7 weeks of gestation); and, *post term* (42 0/7 weeks of gestation and beyond). The American College of Obstetricians and Gynaecologists (ACOG) and the Society for Maternal–Foetal Medicine (SMFM) has endorsed this recommendation and encourages its use for categorising GA [2], [3], [4], [5].

#### 1.1.1 Pathophysiology of preterm birth

Causes of preterm birth are complex and the pathophysiology that triggers preterm birth is largely unknown, however, contributing maternal, foetal and placental predisposing factors have been identified. The most common of these include: antepartum haemorrhage or abruption; mechanical factors such as uterine over-distention and cervical incompetence; hormonal changes; and, bacterial infection and inflammation [6], [7].

Over the past 20 years the access to assisted reproduction technology (ART) in many high income countries has contributed to the rise in the number of multiple births and an overall increase in the rates of preterm delivery. Infants born from multiple pregnancies are more likely to be born preterm due to spontaneous labour or premature rupture of membranes (PROM), or as a result of maternal conditions such as pre-eclampsia or foetal disorders [8], [9]. Changes to policies which limit the number of embryos implanted as part of ART have led to a decline in the number of preterm births due to assisted fertility [10], [11].

Epidemiologic studies have identified preterm birth risk factors as maternal age of less than 17 years or more than 35 years, being underweight, having an overweight pre-pregnancy body mass index, and short stature. Preterm birth rates vary geographically and within ethnic origins, with LMIC consistently having higher rates [7], [12]. Physical and psychosocial stress and smoking have also been associated with higher preterm risk as does a previous preterm birth.

The assessment and diagnosis of preterm birth has remained problematic since it is not a defined disease and the WHO definition does not contain universally recognised reference standards. Different methodologies are used for assessing GA and because reporting rates vary widely between and within countries, accurate comparison of reporting rates of preterm birth and trending data is difficult to analyse [13], [14], [15], [16], [17].

#### 1.1.2 Preterm birth categorisation

Preterm birth defined as less than 37 completed weeks encompasses a wide gestational age range with rates varying across countries. The WHO subcategories of ‘extremely preterm’, ‘very preterm’ and ‘moderate or late preterm’ are recommended to improve comparability of preterm birth data in relation to immunisation.

A limitation of the WHO definition is that there is no boundary between spontaneous abortion and a viable birth, complicating the assessment of preterm birth in the extremely preterm group of babies. A comparison between and within countries becomes complex with varying gestational lower limits of viability over time and across different settings. Determining a lower limit is complex as it is variably defined and arbitrary. It is often described in terms of risk factors and its causes, and is predominately developed according to postnatal viability and data quality in different settings [17], [18], [19], [20].

Preterm births are reported only for live born infants. The pregnancy outcomes differ across countries where the upper limit for national or regional criteria for registration of a foetal death range from 16 weeks to 28 weeks, this impacting on the proportion of preterm births [21].

The registrations of births in LMIC often do not routinely record GA and the data on birthweight (BW) is often not recorded or compiled. It has been reported that 58% of babies in these countries are not weighed at birth and home based births are not represented [20], [22], [23].

#### 1.1.3 Preterm birth following immunisation: what is known in literature?

Pregnant women are at increased risk of morbidity and mortality and adverse pregnancy outcomes, including preterm birth, due to vaccine preventable diseases. Vaccination in pregnancy is a recognised preventive measure for protecting the mother, foetus and infant [24], [25], [26], [27].

Until the 1960s vaccines, including polio, influenza, diphtheria and tetanus toxoid vaccines, were routinely administered to pregnant women in maternal immunisation programmes. Studies in a variety of developed settings detected no increase in adverse consequences for the mother or foetus in vaccinated women [28], [29]. However, the thalidomide teratogenicity disaster in pregnant women resulted in widespread concerns about the safety of all medicine use in pregnancy, including vaccines. Vaccines were then recommended to be only administered in the third trimester of pregnancy to prevent any attribution of teratogenicity risk, as well as to minimise the potential risk to the course of normal gestation such as induction of premature labour [30], [31].

Over recent decades, with further development of safe and immunogenic vaccines, as well as improved ability to explore pregnancy outcome datasets, ongoing studies have provided important information on vaccine safety. Immunisation with inactivated vaccines and toxoids during pregnancy has not been associated with any increased risk to the mother or baby. The extensive use of Tetanus Toxoid (TT) and Tetanus diphtheria (Td) in pregnant women, to prevent neonatal tetanus, has shown no clinically significant adverse events and no adverse pregnancy outcomes for women who have received the Tetanus, diphtheria, acellular pertussis (Tdap) vaccine during pregnancy [32], [33], [34].

The US Advisory Committee on Immunisation Practices (ACIP) in 2012 updated their recommendations to providers of prenatal care to implement a Tdap immunisation programme for all pregnant women to reduce the burden of pertussis in infants. Its recommendation is for its use with every pregnancy. Similarly, in October 2012, the United Kingdom Department of Health recommended a temporary Tdap programme in pregnancy in response to an outbreak [35], [36], [37], [38]. An observational cohort study linking more than 20,000 vaccinated women with pregnancy outcomes showed no increase in stillbirth or other major complications, including preterm birth [39]. Immunisation of pregnant women with inactivated trivalent influenza vaccine has also been recommended and endorsed for more than a decade showing no increase in adverse events. Pregnant women who received H1N1 influenza vaccine during the 2009 H1N1 influenza pandemic were in fact less likely to give birth preterm [40], [41], [42], [43], [44], [45], [46], [47].

Live viral vaccines, such as measles, mumps, rubella (MMR); varicella; intranasal live-attenuated influenza; Yellow Fever, and BCG however are contraindicated and not recommended during pregnancies, with a theoretical risk that the vaccine virus could be transmitted to the foetus [48]. Follow up of inadvertent vaccinations of pregnant women with live vaccines have not demonstrated significant adverse effects but these limited data have not been sufficient to change recommendations. The risk benefit to the mother and neonate needs to be taken into account.

#### 1.1.4 Existing case definitions for preterm birth

Historically, preterm birth was determined using neonatal physical examination, reviewing clinical history and socio-demographics [49], [50]. Early definitions of prematurity relied on BW, using a birth weight category of less than 2300 or 2500 g. One of the earliest working definitions was introduced by the World Health Assembly (WHA) in 1948 using a birth weight of 2500 g (5 pounds, 8 ounces) or less as a determinant [49].

Early epidemiological studies of prematurity tended to include all low birth weight babies irrespective of gestation. BW was used as a criterion alone as it was objective, easily measured, and the survival of the very low birth weight (VLBW) neonates was described in birth weight specific categories [51], [52], [53], [54]. BW however, can only be used as a surrogate in the lower gestational age babies where it has been identified to be a specific and sensitive method in assessing the early preterm. Babies weighing less than 1500 g are predominately assessed as being preterm [55], [56]. The lack of standardised birthweight categories makes it difficult to analyse and compare data from different regions [22], [57], [58], [59].

However, BW based standards for preterms are complicated by physiological variables that occur more commonly pregnancies complicated by preterm birth [60], [61]. Preterm infants are often growth restricted and conventional BW charts are limited as they do not reflect the degree of growth restriction. The Foetal Growth Longitudinal Study (FGLS), part of the INTERGROWTH-21 Project developed international growth and size standards for foetuses. The growth standards are recommended for the clinical interpretation of ultrasound measurements and for comparisons across populations [62]. Foetal growth standards for preterm infants will determine the precise incidence of foetal growth restriction when gestation is known [63], [64], [65].

The World Health Organisation (WHO) definition of preterm birth remains the most widely utilised and accepted definition. The International Classification of Diseases (ICD-9, ICD-10) defines preterm birth as less than 37 completed weeks (less than 259 days) of gestation. The duration of gestation is measured from the first day of the LMP. GA is expressed in completed days or completed weeks (e.g., events occurring 280–286 completed days after the onset of the LMP are considered to have occurred at 40 weeks of gestation). Where the date of the LMP is not available, GA is based on the best clinical estimate [2].

A search of terminology databases, including the Global Alliance on Prevention of Prematurity (GAPPS), the National Institutes of Health, and the Common Terminology Criteria for Adverse Events (CTCAE) show a consistency with the WHO definition as the onset of labour before 37 completed weeks of pregnancy (full term is 40 completed weeks) [7], [66].

Essential in any definition or the sub classification of preterm birth is the need for accurate dating. GA has evolved as the stand alone parameter for determining preterm birth. In 1949 the U.S. National Centre for Health Statistics of the Centres for Disease Control and Prevention revised the World Health Assembly definition and deleted the reference to BW, to include only the reporting of the length of pregnancy in weeks. In 1956 this was further revised to specify the reporting of completed weeks of gestation [67].

A 1961 report by the Expert Committee on Maternal and Child Health of the World Health Organisation highlighted the difference between premature and those infants of low birth weight (LBW) and in 1970 a working party of obstetricians and paediatricians at the Second European Congress of Perinatal Medicine set the boundary between preterm and term birth at 37 weeks of gestation [5].

This is the basis of the most recent widely utilised and accepted definition of prematurity [68].

Before the development of more accurate methods of estimating gestation, the LMP remains as the most widely available measure. This method is used where ultrasound (US) is not available or accessible and is recommended by the WHO for determining preterm birth [12], [69], [70].

When available, in the clinical context, it is a valid and applicable measure, especially when estimating gestation of less than or equal to 33 weeks. Its limitations are discussed as being a determinant based on self-reporting and therefore felt to be imprecise. Studies have shown, however, that women who were certain of their LMP were accurate in their assessment of pregnancy duration compared with their ultrasound dating [71].

When information about the LMP is absent or uncertain, estimates of gestational age can be determined from a clinical assessment including the description of pregnancy symptoms such as nausea, fatigue, tender swollen breasts, frequent urination, a pelvic examination when performed in the first trimester and fundal height (FH) ascertainment. One study demonstrated a high correlation between early pregnancy dating up to 9 completed weeks by a clinician based on an examination and history and that determined by ultrasound but this is influenced by the skill and experience of the clinician [72].

Fundal height (FH) is often used in conjunction with LMP and/or the BW of the neonate, especially in low resource settings. Women from many traditional societies however often do not record their LMP date and can present late in the first trimester. Variability in FH measurements relate to previous caesarean section (C/S), multiple pregnancy, race, maternal height, intrauterine growth retardation, maternal obesity, polyhydramnios, and a difference in examination techniques. FH measurement is not standardised and use beyond 16 weeks reduces in accuracy, affecting its reliability and the precision of dating [58], [73], [74]. Its use in combination with more sensitive assessment methods is recommended.

Through the advent of US, the use of early home pregnancy tests, ART such as intrauterine insemination (IUI), and home ovulation test kits, the actual timing of conception can be determined and therefore accurate dating of gestational age performed. Pregnancies achieved through ART represent the most accurate method.

Outside the use of ART, an ultrasound performed in the first trimester (≤13 6/7 weeks), is viewed as the most accurate and reliable measure. There has been a shift from using LMP to using US for predicting an actual date of delivery. Estimation of the foetal crown rump length ± biparietal diameter/femur length between the gestational age of 6–18 weeks shows an accuracy within 5–7 days. In women with uncertain dates an early US is recommended for optimal dating [3].

The methodology for US gestational age assessment, however, is not standardised and tends to give a transitory increase in preterm births when compared to the use of LMP alone. In addition, US accessibility in LMIC is limited for the majority of women and therefore cannot be considered a universal measure for determining preterm birth [20], [57], [75], [76], [77], [78]. Defining preterm delivery for LMIC would therefore be strengthened by the use of one or both measures when available [4], [79], [80], [81], [82], [83].

Where measurements such as LMP, US or antenatal clinical assessment are absent or likely to be inaccurate, a recommended criterion for determining preterm birth is a clinical estimation of gestational age based on the physical and neurological examination of the neonate [84], [85]. A review of methods used identified tools based on neurological and physical criteria, or physical criteria alone. Methods that use neurologic criteria are proven, reliable measures with expert operators but the feasibility for use is compromised especially in LMIC, being limited by complexity, and requiring skill and experience to perform [70], [86], [87], [88], [89]. Ascertainment of neurologic signs and external characteristics used in the assessment need to be precise and accurate to ensure correct correlation with actual GA [51], [90], [91].

The physical examination based systems have been refined and modified to improve their applicability and accuracy. Some methods now use external characteristics alone, enabling gestational age to be determined and estimated in all settings. Using the available methods ranging in the current criteria, there is a correlation with LMP based estimation of over 90%, but an acknowledged range of error for predicting clinical maturity of ±2.4 weeks. Physical examination tools alone, when used for neonates under 28–33 weeks are recognised to be inaccurate and are therefore are not recommended to be used as a measure to accurately estimate the gestation of neonates within the lower limit of viability [92], [93], [94], [95], [96], [97].

The Ballard Maturational Score, known as the New Ballard Score, uses both physical and neurological assessment and has been refined and expanded to include extremely premature neonates and is described as a valid and accurate gestational assessment tool [98].

### 1.2 Methods for the development of the case definition and guidelines for data collection, analysis, and presentation for preterm birth as an adverse events following immunisation

Following the process described in the overview paper as well as on the Brighton Collaboration Website http://www.brightoncollaboration.org/internet/en/index/process.html, the Brighton Collaboration Preterm Birth Working Group was formed in 2015 and included members of clinical, academic, public health, and industry background. The composition of the working and reference group as well as results of the web-based survey completed by the reference group with subsequent discussions in the working group can be viewed at: http://www.brightoncollaboration.org/internet/en/index/workinggroups.html.

To guide the decision-making for the case definition and guidelines, a literature search was performed using Medline, Embase, PubMed, Cinahl, Clincal Key and the Cochrane Libraries for published work relevant to this review with the search terms including: ‘prematurity’; ‘preterm birth’; ‘neonatal outcomes’; ‘birth outcomes’; ‘gestational age’; ‘premature labour’; ‘preterm delivery’; ‘spontaneous labour’; ‘antenatal’; ‘low birth weight’; ‘neonatal mortality’; ‘stillbirth’; ‘extremely preterm’; ‘moderate preterm’; ‘late preterm’; ‘neurological assessment’; ‘vaccination’; ‘immunisation’; ‘viability’; ‘spontaneous labour’; ‘epidemiology’; ‘perinatal outcomes’; ‘last menstrual period’; ‘LMP’; ‘ultrasound’; ‘US’; ‘pregnancy duration’; ‘obstetrics’; ‘morbidity’; ‘small for gestational age’; ‘birthweight’; ‘foetal growth’; ‘multiple pregnancy’; ‘pregnancy’; ‘risk factors’; ‘fundal height’; and terms in combination. We focused on work published published in English language, but also included commonly referenced older publications. The search resulted in the identification of references, 262 articles with potentially relevant material were reviewed in more detail in order to identify studies using case definitions or, in their absence, providing clinical descriptions of the case material. This review resulted in a detailed summary of 99 articles, including information on the diagnostic criteria or case definition put forth.

### 1.3 Rationale for selected decisions about the case definition of preterm birth as an adverse event following immunisation

An ideal standardised case definition aims to improve reliability and comparability of data collected from immunised mothers that deliver full term or preterm infants across all health care settings. A functional case definition is important for the evaluation and assessment of data to help determine whether a vaccine administered during pregnancy may or may not be implicated in a subsequent preterm birth. The definition must be applicable in regions that are geographically and administratively diverse, regardless of available health care personnel, training and resources.

There has never been a single unanimously accepted definition of prematurity. The literature identified was notable for inconsistent definitions and numerous descriptions for preterm birth. Existing definitions categorise preterm birth by clinical presentation, BW and GA.

There is hence no uniformly accepted definition of preterm birth that is able to be employed to evaluate its occurrence following antenatal immunisations. This poses a missed opportunity and a potential risk to immunisation trials and programmes as well as pregnant women, as potential associations may be falsely raised or dismissed without an agreed case definition. Data comparability across a myriad of trials and varying surveillance systems would facilitate data interpretation and improve the ability of investigators and regulators to confidently detect or rule out any association between immunisations in pregnancy and preterm birth. The work group recommended the use of the current definition for Preterm Birth with the subcategories of extremely preterm, very preterm and moderate or late preterm. Accurate dating is essential for the definition, with GA assessment criteria implicit for categorising.

#### 1.3.1 Formulating a case definition that reflects diagnostic certainty: weighing specificity versus sensitivity

It needs to be re-emphasised that the grading of definition levels is entirely about diagnostic certainty of the event, not its clinical severity. Thus, a very severe clinical event, like Preterm Birth, may appropriately be classified as Level Two or Three rather than Level One, based upon the level confidence the birth truly occurred preterm.

The amount of information, including number of symptoms and/or signs, that will be documented for each case may vary considerably. The case definition has been formulated such that the Level 1 definition is highly specific (or confident) for preterm birth. As maximum specificity normally implies a loss of sensitivity, two additional diagnostic levels have been included in the definition, offering a stepwise increase of sensitivity from Level One down to Level Three, while retaining an acceptable level of specificity at all levels. In this way it is hoped that all possible cases of Preterm Birth can be captured.

#### 1.3.2 Precision of gestational age assessment

The most accurate methods for GA assessment are included in Level 1 of diagnostic certainty. The GAIA Preterm Birth working group considered that pregnant women with a certain menstrual date or those who have undergone IUI or Embryo Transfer (ET) with a confirmatory 1st trimester scan (≤13 6/7 weeks) or a 1st trimester ultrasound established date (≤13 6/7 weeks) alone, as representing the “gold standard” of diagnostic certainty of GA assessment, and therefore the likelihood of preterm birth. If the LMP date and U/S date do not correlate, defaulting to U/S for GA assessment is required.

Where there is no 1st trimester scan or ART performed, Level 2A of diagnostic certainty is used to describe gestational dating. With a certain menstrual date, either a confirmatory 2nd trimester ultrasound established date (14 0/7 weeks to 27 6/7 weeks) or a 1st trimester pelvic bimanual examination are considered the next most precise measurement methodologies. Where there is no menstrual date, it is recommended that the 2nd trimester ultrasound established date be used and categorised as Level 2B.

Level 3A of diagnostic certainty represents a certain menstrual date with a 3rd trimester scan of 28 0/7 weeks+, or a confirmatory 2nd trimester fundal height, or birth weight. Where there is no menstrual date, a 1st trimester pelvic bimanual examination would meet the requirement. A separate level 3B category was recommended where there is an uncertain or no menstrual date. In this case a fundal height, or newborn physical assessment or birth weight would meet the requirement for this level.

##### 1.3.2.1 Timing post immunisation in pregnancy

In the absence of existing data supporting an association between any vaccine and preterm birth, the working group did not feel it was appropriate to define a ‘risk window’ following vaccination during pregnancy and subsequent possible Preterm Birth. The case definition of the outcome (Preterm Birth) is independent from the exposure (e.g., immunisations). Therefore, to avoid selection bias, a restrictive time interval from immunisation to onset of Preterm Birth has not been included. Instead, where feasible, details of this interval should be assessed and reported as described in the data collection guidelines.

Further, Preterm Birth often occurs outside the controlled settings of clinical trials or hospitals. In some settings it may be impossible to obtain a clear timeline of the event, particularly in less developed or rural settings. In order to avoid excluding such cases, the Brighton Collaboration case definition avoids setting arbitrary time frames.

### 1.4 Guidelines for data collection, analysis and presentation

As mentioned in the overview paper, the case definition is accompanied by guidelines which are structured according to the steps of conducting a clinical trial, i.e. data collection, analysis and presentation. Neither case definition nor guidelines are intended to guide or establish criteria for management of ill infants, children, or adults. Both were developed to improve data comparability.

### 1.5 Periodic review

Similar to all Brighton Collaboration case definitions and guidelines, review of the definition with its guidelines is planned on a regular basis (i.e. every three to five years) or more often if needed.

## 2 Case definition of preterm birth

### 2.1 Prematurity and assessment of gestational age criteria

  **Definitions of terms used:**

• **Intrauterine insemination (IUI)** – A procedure in which a fine catheter is inserted through the cervix into the uterus to deposit a sperm sample directly into the uterus, to achieve fertilisation and pregnancy.
• **Embryo transfer –** The procedure in which one or more embryos are placed in the uterus or fallopian tube.
• ***Ultrasound (U/S)*** [62]**:**
- 1st trimester (≤13 6/7 weeks).
- 2nd trimester scan (14 0/7–27 6/7 weeks).
- 3rd trimester (28 0/7 + weeks).
• **LMP (last menstrual period) –** Gestational age is calculated from the first day of the mother's last menstrual period.

  ***If LMP and U/S do not correlate, default to U/S GA assessment***

*Certain LMP: (LMP date + 280 days): Use LMP if within 7 days at ≤14 weeks; within 14 days at ≤26 weeks; within 21 days beyond 26 weeks.

*Uncertain LMP – first trimester (≤13 6/7 weeks by LMP): Use the approximate date of the last menstrual period (LMP) if corroborated by physical exam, or a first trimester ultrasound. If there is a discrepancy of >7 days between the LMP and the first trimester ultrasound, the ultrasound-established dates will take preference over LMP for gestational age dating.

*Uncertain LMP – second trimester (14 0/7–27 6/7 weeks by LMP): Use the approximate date of the LMP if corroborated by physical exam including fundal height, or a second trimester ultrasound. If there is a discrepancy of >10 days between the LMP and the second trimester ultrasound, the ultrasound-established dates will take preference over LMP for gestational age dating.

*Uncertain LMP – third trimester >28 weeks – third trimester ultrasound.

*No LMP date: If menstrual dates are unknown, the ultrasound-established dates will be used for gestational age dating or 2nd trimester fundal height and/or newborn physical examination.

• **Pregnancy symptoms**– nausea, fatigue, tender swollen breasts, frequent urination.
• **Antenatal Physical Examination**– pelvic bimanual examination confirming enlarged uterus [63].
• **Newborn Physical Examination– New Ballard Score** – physical and neurological assessment – Appendix 1.
• **Fundal Height(FH) in cms** – Appendix 2.
• **Birth Weight (BW) in grams** – Appendix 2.

### 2.2 Prematurity and assessment of gestational age

  ***Level 1: (highest level of certainty)***

**1.** Certain LMP* or intrauterine insemination (IUI) date or embryo transfer (ET) date ***with*** **confirmatory 1st trimester scan (≤13 6/7 weeks).**
  **OR**
**2.** **1st trimester scan (≤13 6/7 weeks).**

  ***Level 2A***

**1.** Certain LMP* with 2nd trimester scan (14 0/7 weeks to 27 6/7 weeks). If LMP and U/S do not correlate, default to U/S GA assessment.
  **OR**
**2.** Certain LMP* ***with*** **1st trimester physical examination.**

  ***Level 2B***

**Uncertain LMP with 2nd trimester scan (14 0/7 weeks to 27 6/7 weeks).**

  ***Level 3A***

**1.** **Certain LMP** ***with*** **3rd trimester scan – 28 0/7 weeks +.**
  **OR**
**2.** **Certain LMP** ***with*** **confirmatory 2nd trimester FH.**
  **OR**
**3.** **Certain LMP with birth weight.**
  **OR**
**4.** **Uncertain LMP with 1st trimester physical examination.**

  ***Level 3B***

**1.** **Uncertain LMP** ***with*** **FH.**
  **OR**
**2.** **Uncertain LMP** ***with*** **newborn physical assessment.**
  **OR**
**3.** **Uncertain LMP** ***with*** **Birth weight.**

## 3 Guidelines for data collection, analysis and presentation of preterm birth

It was the consensus of the Brighton Collaboration Working Group for Preterm Birth to recommend the following guidelines to enable meaningful and standardised collection, analysis, and presentation of information about Preterm Birth. However, implementation of all guidelines might not be possible in all settings. The availability of information may vary depending upon resources, geographical region, and whether the source of information is a prospective clinical trial, a post-marketing surveillance or epidemiological study, or an individual report of Preterm Birth. Also, as explained in more detail in the overview paper in this volume, these guidelines have been developed by this working group for guidance only, and are not to be considered a mandatory requirement for data collection, analysis, or presentation.

### 3.1 Data collection

These guidelines represent a desirable standard for the collection of data on availability following immunisation to allow for comparability of data, and are recommended as an addition to data collected for the specific study question and setting. The guidelines are not intended to guide the primary reporting of Preterm Birth to a surveillance system or study monitor. Investigators developing a data collection tool based on these data collection guidelines also need to refer to the criteria in the case definition, which are not repeated in these guidelines.

Guidelines numbers below have been developed to address data elements for the collection of adverse event information as specified in general drug safety guidelines by the International Conference on Harmonization of Technical Requirements for Registration of Pharmaceuticals for Human Use [99], and the form for reporting of drug adverse events by the Council for International Organisations of Medical Sciences [100]. These data elements include an identifiable reporter and patient, one or more prior immunisations, and a detailed description of the adverse event, in this case, Preterm Birth following maternal immunisation. The additional guidelines have been developed as guidance for the collection of additional information to allow for a more comprehensive understanding of Preterm Birth following maternal immunisation.

#### 3.1.1 Source of information/reporter

For all cases and/or all study participants, as appropriate, the following information should be recorded:

(1) Date of report.
(2) Name and contact information of person reporting^2^ and/or diagnosing the Preterm Birth as specified by country-specific data protection law.
(3) Name and contact information of the investigator or clinician responsible for the subject, as applicable.
(4) Relation to the patient (e.g., immuniser [clinician, nurse], family member [indicate relationship], other).

#### 3.1.2 Vaccinee/control

##### 3.1.2.1 Demographics

For all cases and/or all study participants, as appropriate, the following information should be recorded:

(5) Case/study participant identifiers (e.g., first name initial followed by last name initial) or code (or in accordance with country-specific data protection laws).
(6) Date of birth, age, and sex.
(7) For infants: gestational age and birth weight.

##### 3.1.2.2 Clinical and immunisation history

For all cases and/or all study participants, as appropriate, the following information regarding the immunised woman should be recorded:

(8) Past medical history, including hospitalisations, underlying diseases/disorders, pre-immunisation signs and symptoms including identification of indicators for, or the absence of, a history of allergy to vaccines, vaccine components or medications; food allergy; allergic rhinitis; eczema; asthma.
(9) Any medication history (other than treatment for the event described) prior to, during, and after immunisation including prescription and non-prescription medication as well as medication or treatment with long half-life or long term effect (e.g., immunoglobulins, blood transfusion and immunosuppressants).
(10) Immunisation history (i.e. previous immunisations and any adverse event following immunisation (AEFI)), in particular occurrence of Preterm Birth after a previous immunisation.

#### 3.1.3 Details of the immunisation

For all cases and/or all study participants, as appropriate, the following information should be recorded:

(11) Date and time of maternal immunisation(s).
(12) Description of vaccine(s) (name of vaccine, manufacturer, lot number, dose (e.g., 0.25 mL, 0.5 mL, etc.) and number of dose if part of a series of immunisations against the same disease).
(13) The anatomical sites (including left or right side) of all immunisations (e.g., vaccine A in proximal left lateral thigh, vaccine B in left deltoid).
(14) Route and method of administration (e.g., intramuscular, intradermal, subcutaneous, and needle-free (including type and size), other injection devices).
(15) Needle length and gauge.

#### 3.1.4 The adverse event

(16) For all cases at any level of diagnostic certainty and for reported events with insufficient evidence, the criteria fulfilled to meet the case definition should be recorded.

**Specifically document**

(17) Clinical description of signs and sympt oms of Preterm Birth in immunised woman and newborn and if there was medical confirmation of the event (i.e. patient seen by physician).
(18) Date/time of onset,^3^ first observation^4^ and diagnosis,^5^ end of episode^6^ and final outcome.^7^
(19) Concurrent signs, symptoms, and diseases in immunised woman and newborn.
(20) Measurement/testing
• Values and units of routinely measured parameters (e.g., temperature, blood pressure) – in particular those indicating the severity of the event.
• Method of measurement (e.g., type of thermometer, oral or other route, duration of measurement, etc.).
• Results of laboratory examinations, surgical and/or pathological findings and diagnoses if present.
(21) Treatment given for Preterm Birth to mother and/or newborn, especially specify what medication and dosing, or specific interventions.
(22) Outcome^8^ at last observation.
(23) Objective clinical evidence supporting classification of the event as “serious”.^9^
(24) Exposures other than the immunisation 24 h before and after immunisation (e.g., food, environmental) considered potentially relevant to the reported event.

#### 3.1.5 Miscellaneous/general

(25) The duration of surveillance for Preterm Birth should be until the pregnancy has been completed, but specific surveillance may be further predefined based on
• Biologic characteristics of the vaccine e.g., live attenuated versus inactivated component vaccines.
• Biologic characteristics of the vaccine-targeted disease.
• Biologic characteristics of Preterm Birth including patterns identified in previous trials (e.g., early-phase trials).
• Biologic characteristics of the vaccinee (e.g., nutrition, underlying disease like immunodepressing illness).
(26) The duration of follow-up reported during the surveillance period should be predefined likewise. It should aim to continue to resolution of the event.
(27) Methods of data collection should be consistent within and between study groups, if applicable.
(28) Follow-up of cases should attempt to verify and complete the information collected as outlined in data collection guidelines 1–24.
(29) Investigators of patients with Preterm Birth should provide guidance to reporters to optimise the quality and completeness of information provided.
(30) Reports of Preterm Birth should be collected throughout the study period regardless of the time elapsed between immunisation and the adverse event. If this is not feasible due to the study design, the study periods during which safety data are being collected should be clearly defined.

### 3.2 Data analysis

The following guidelines represent a desirable standard for analysis of data on Preterm Birth to allow for comparability of data, and are recommended as an addition to data analysed for the specific study question and setting.

(31) Reported events should be classified in one of the following five categories including the three levels of diagnostic certainty. Events that meet the case definition should be classified according to the levels of diagnostic certainty as specified in the case definition. Events that do not meet the case definition should be classified in the additional categories for analysis.

Event classification in 5 categories^10^

Event meets case definition

(1) Level 1: Criteria as specified in the Preterm Birth case definition.
(2) Level 2: Criteria as specified in the Preterm Birth case definition.
(3) Level 3: Criteria as specified in the Preterm Birth case definition.

Event does not meet case definition

*Additional categories for analysis*

(4) Reported Preterm Birth with insufficient evidence to meet the case definition.^11^
(5) Not a case of Preterm Birth.

(32) The interval between immunisation and reported Preterm Birth could be defined as the date/time of immunisation to the date/time of onset^12^ of the newborn delivery. If few cases are reported, the concrete time course could be analysed for each; for a large number of cases, data can be analysed in the following increments:

**Subjects with preterm birth by interval to presentation**

**Table:** 

Interval*	Number
<1 week after immunisation	
1–<2 weeks after immunisation	
2–<4 weeks after immunisation	
4–<6 weeks after immunisation	
4 week increments thereafter	
Total

(33) The duration of a possible Preterm Birth could be analysed as the interval between the date/time of onset^1^ of the first symptoms and/or signs consistent with the definition and the end of episode^5^ and/or final outcome.^6^ Whatever start and ending are used, they should be used consistently within and across study groups.
(34) If more than one measurement of a particular criterion is taken and recorded, the value corresponding to the greatest magnitude of the adverse experience could be used as the basis for analysis. Analysis may also include other characteristics like qualitative patterns of criteria defining the event.
(35) The distribution of data (as numerator and denominator data) could be analysed in predefined increments (e.g., measured values, times), where applicable. Increments specified above should be used. When only a small number of cases is presented, the respective values or time course can be presented individually.
(36) Data on Preterm Birth obtained from subjects receiving a vaccine should be compared with those obtained from an appropriately selected and documented control group(s) to assess background rates of Preterm Birth in non-exposed populations, and should be analysed by study arm and dose where possible, e.g., in prospective clinical trials.

### 3.3 Data presentation

These guidelines represent a desirable standard for the presentation and publication of data on Preterm Birth following maternal immunisation to allow for comparability of data, and are recommended as an addition to data presented for the specific study question and setting. Additionally, it is recommended to refer to existing general guidelines for the presentation and publication of randomised controlled trials, systematic reviews, and meta-analyses of observational studies in epidemiology (e.g., statements of Consolidated Standards of Reporting Trials (CONSORT), of Improving the quality of reports of meta-analyses of randomised controlled trials (QUORUM), and of Meta-analysis Of Observational Studies in Epidemiology (MOOSE), respectively [101], [102], [103]).

(37) All reported events of Preterm Birth should be presented according to the categories listed in guideline 31.
(38) Data on possible Preterm Birth events should be presented in accordance with data collection guidelines 1–24 and data analysis guidelines 31–36.
(39) Terms to describe Preterm Birth such as “low-grade”, “mild”, “moderate”, “high”, “severe” or “significant” are highly subjective, prone to wide interpretation, and should be avoided, unless clearly defined.
(40) Data should be presented with numerator and denominator (*n*/*N*) (and not only in percentages), if available.

Although immunisation safety surveillance system denominator data are usually not readily available, attempts should be made to identify approximate denominators. The source of the denominator data should be reported and calculations of estimates be described (e.g., manufacturer data like total doses distributed, reporting through Ministry of Health, coverage/population based data, etc.).

(41) The incidence of cases in the study population should be presented and clearly identified as such in the text.
(42) If the distribution of data is skewed, median and range are usually the more appropriate statistical descriptors than a mean. However, the mean and standard deviation should also be provided.
(43) Any publication of data on Preterm Birth should include a detailed description of the methods used for data collection and analysis as possible. It is essential to specify:
• The study design.
• The method, frequency and duration of monitoring for Preterm Birth.
• The trial profile, indicating participant flow during a study including drop-outs and withdrawals to indicate the size and nature of the respective groups under investigation.
• The type of surveillance (e.g., passive or active surveillance).
• The characteristics of the surveillance system (e.g., population served, mode of report solicitation).
• The search strategy in surveillance databases.
• Comparison group(s), if used for analysis.
• The instrument of data collection (e.g., standardised questionnaire, diary card, report form).
• Whether the day of immunisation was considered “day one” or “day zero” in the analysis.
• Whether the date of onset^2^ and/or the date of first observation^3^ and/or the date of diagnosis^4^ was used for analysis.
• Use of this case definition for Preterm Birth, in the abstract or methods section of a publication.^11^

## Footnotes

2 If the reporting centre is different from the vaccinating centre, appropriate and timely communication of the adverse event should occur.

3 The date and/or time of onset is defined as the time post immunisation, when the first sign or symptom indicative for Preterm Birth occurred. This may only be possible to determine in retrospect.

4 The date and/or time of first observation of the first sign or symptom indicative for Preterm Birth can be used if date/time of onset is not known.

5 The date of diagnosis of an episode is the day post immunisation when the event met the case definition at any level.

6 The end of an episode is defined as the time the event no longer meets the case definition at the lowest level of the definition.

7 E.g., recovery to pre-immunisation health status, spontaneous resolution, therapeutic intervention, persistence of the event, sequelae, death.

8 An AEFI is defined as serious by international standards if it meets one or more of the following criteria: (1) it results in death, (2) is life-threatening, (3) it requires inpatient hospitalisation or results in prolongation of existing hospitalisation, (4) results in persistent or significant disability/incapacity, (5) is a congenital anomaly/birth defect, (6) is a medically important event or reaction.

9 To determine the appropriate category, the user should first establish, whether a reported event meets the criteria for the lowest applicable level of diagnostic certainty, e.g., Level three. If the lowest applicable level of diagnostic certainty of the definition is met, and there is evidence that the criteria of the next higher level of diagnostic certainty are met, the event should be classified in the next category. This approach should be continued until the highest level of diagnostic certainty for a given event could be determined. Major criteria can be used to satisfy the requirement of minor criteria. If the lowest level of the case definition is not met, it should be ruled out that any of the higher levels of diagnostic certainty are met and the event should be classified in additional categories four or five.

10 If the evidence available for an event is insufficient because information is missing, such an event should be categorised as “Reported Preterm Birth with insufficient evidence to meet the case definition”.

11 An event does not meet the case definition if investigation reveals a negative finding of a necessary criterion (necessary condition) for diagnosis. Such an event should be rejected and classified as “Not a case of Preterm Birth”.

12 Use of this document should preferably be referenced by referring to the respective link on the Brighton Collaboration website (http://www.brightoncollaboration.org).
