# Supplementary material for: Preterm birth: Case definition & guidelines for data collection, analysis, and presentation of immunisation safety data
Source: Vaccine. 2016 Dec 1;34(49):6047–56. doi: 10.1016/j.vaccine.2016.03.045 (PMC5139808; doi:10.1016/j.vaccine.2016.03.045)
Supplement: Supplementary file 1 [file mmc1.docx]

**APPENDIX 1:**

**New Ballard Score Sheet**

**NEUROMUSCULAR MATURITY**

| SIGN | SCORE | | | | | | | SIGN SCORE |
| --- | --- | --- | --- | --- | --- | --- | --- | --- |
|  | -1 | 0 | 1 | 2 | 3 | 4 | 5 |  |
| Posture |  |  |  |  |  |  |  |  |
| Square Window |  |  |  |  |  |  |  |  |
| Arm Recoil |  |  |  |  |  |  |  |  |
| Popliteal Angle |  |  |  |  |  |  |  |  |
| Scarf Sign |  |  |  |  |  |  |  |  |
| Heel To Ear |  |  |  |  |  |  |  |  |
| TOTAL NEUROMUSCULAR SCORE | | | | | | | |  |

**PHYSICAL MATURITY**

| SIGN | SCORE | | | | | | | SIGN SCORE |
| --- | --- | --- | --- | --- | --- | --- | --- | --- |
|  | -1 | 0 | 1 | 2 | 3 | 4 | 5 |  |
| Skin | [Sticky, friable, transparent](javascript:__doPostBack('ctl00$ContentPlaceHolder1$SkinButtonMin1','')) | [gelatinous, red, translucent](javascript:__doPostBack('ctl00$ContentPlaceHolder1$SkinButton0','')) | [smooth pink, visible veins](javascript:__doPostBack('ctl00$ContentPlaceHolder1$SkinButton1','')) | [superficial peeling &/or rash, few veins](javascript:__doPostBack('ctl00$ContentPlaceHolder1$SkinButton2','')) | [cracking, pale areas, rare veins](javascript:__doPostBack('ctl00$ContentPlaceHolder1$SkinButton3','')) | [parchment, deep cracking, no vessels](javascript:__doPostBack('ctl00$ContentPlaceHolder1$SkinButton4','')) | [leathery, cracked, wrinkled](javascript:__doPostBack('ctl00$ContentPlaceHolder1$SkinButton5','')) |  |
| Lanugo | [none](javascript:__doPostBack('ctl00$ContentPlaceHolder1$LanugoButtonMin1','')) | [sparse](javascript:__doPostBack('ctl00$ContentPlaceHolder1$LanugoButton0','')) | [abundant](javascript:__doPostBack('ctl00$ContentPlaceHolder1$LanugoButton1','')) | [thinning](javascript:__doPostBack('ctl00$ContentPlaceHolder1$LanugoButton2','')) | [bald areas](javascript:__doPostBack('ctl00$ContentPlaceHolder1$LanugoButton3','')) | [mostly bald](javascript:__doPostBack('ctl00$ContentPlaceHolder1$LanugoButton4','')) |  |  |
| Plantar Surface | heel-toe [40-50mm: -1](javascript:__doPostBack('ctl00$ContentPlaceHolder1$PlantarButtonMin1',''))  [<40mm: -2](javascript:__doPostBack('ctl00$ContentPlaceHolder1$PlantarButtonMin2','')) | [>50 mm no crease](javascript:__doPostBack('ctl00$ContentPlaceHolder1$PlantarButton0','')) | [faint red marks](javascript:__doPostBack('ctl00$ContentPlaceHolder1$PlantarButton1','')) | [anterior transverse crease only](javascript:__doPostBack('ctl00$ContentPlaceHolder1$PlantarButton2','')) | [creases ant. 2/3](javascript:__doPostBack('ctl00$ContentPlaceHolder1$PlantarButton3','')) | [creases over entire sole](javascript:__doPostBack('ctl00$ContentPlaceHolder1$PlantarButton4','')) |  |  |
| Breast | [imperceptable](javascript:__doPostBack('ctl00$ContentPlaceHolder1$BreastButtonMin1','')) | [barely perceptable](javascript:__doPostBack('ctl00$ContentPlaceHolder1$BreastButton0','')) | [flat areola no bud](javascript:__doPostBack('ctl00$ContentPlaceHolder1$BreastButton1','')) | [stippled areola 1-2 mm bud](javascript:__doPostBack('ctl00$ContentPlaceHolder1$BreastButton2','')) | [raised areola 3-4 mm bud](javascript:__doPostBack('ctl00$ContentPlaceHolder1$BreastButton3','')) | [full areola 5-10 mm bud](javascript:__doPostBack('ctl00$ContentPlaceHolder1$BreastButton4','')) |  |  |
| Eye / Ear | lids fused [loosely: -1](javascript:__doPostBack('ctl00$ContentPlaceHolder1$EarButtonMin1',''))  [tightly: -2](javascript:__doPostBack('ctl00$ContentPlaceHolder1$EarButtonMin2','')) | [lids open pinna flat stays folded](javascript:__doPostBack('ctl00$ContentPlaceHolder1$EarButtonMin0','')) | [sl. curved pinna; soft; slow recoil](javascript:__doPostBack('ctl00$ContentPlaceHolder1$EarButton1','')) | [well-curved pinna; soft but ready recoil](javascript:__doPostBack('ctl00$ContentPlaceHolder1$EarButton2','')) | [formed & firm instant recoil](javascript:__doPostBack('ctl00$ContentPlaceHolder1$EarButton3','')) | [thick cartilage ear stiff](javascript:__doPostBack('ctl00$ContentPlaceHolder1$EarButton4','')) |  |  |
| Genitals (Male) | [scrotum flat, smooth](javascript:__doPostBack('ctl00$ContentPlaceHolder1$MaleButtonMin1','')) | [scrotum empty, faint rugae](javascript:__doPostBack('ctl00$ContentPlaceHolder1$MaleButton0','')) | [testes in upper canal, rare rugae](javascript:__doPostBack('ctl00$ContentPlaceHolder1$MaleButton1','')) | [testes descending, few rugae](javascript:__doPostBack('ctl00$ContentPlaceHolder1$MaleButton2','')) | [testes down, good rugae](javascript:__doPostBack('ctl00$ContentPlaceHolder1$MaleButton3','')) | [testes pendulous, deep rugae](javascript:__doPostBack('ctl00$ContentPlaceHolder1$MaleButton4','')) |  |  |
| Genitals (Female) | [clitoris prominent & labia flat](javascript:__doPostBack('ctl00$ContentPlaceHolder1$FemaleButtonMin1','')) | [prominent clitoris & small labia minora](javascript:__doPostBack('ctl00$ContentPlaceHolder1$FemaleButton0','')) | [prominent clitoris & enlarging minora](javascript:__doPostBack('ctl00$ContentPlaceHolder1$FemaleButton1','')) | [majora & minora equally prominent](javascript:__doPostBack('ctl00$ContentPlaceHolder1$FemaleButton2','')) | [majora large, minora small](javascript:__doPostBack('ctl00$ContentPlaceHolder1$FemaleButton3','')) | [majora cover clitoris & minora](javascript:__doPostBack('ctl00$ContentPlaceHolder1$FemaleButton4','')) |  |  |
| TOTAL PHYSICAL MATURITY SCORE | | | | | | | |  |

Top of Form

**MATURITY RATING TABLE**

| TOTAL SCORE  (NEUROMUSCULAR + PHYSICAL) | WEEKS |
| --- | --- |
|  |  |
| -10 | 20 |
| -5 | 22 |
| 0 | 24 |
| 5 | 26 |
| 10 | 28 |
| 15 | 30 |
