# Supplementary material for: Preterm birth: Case definition & guidelines for data collection, analysis, and presentation of immunisation safety data
Source: Vaccine. 2016 Dec 1;34(49):6047–56. doi: 10.1016/j.vaccine.2016.03.045 (PMC5139808; doi:10.1016/j.vaccine.2016.03.045)
Supplement: Supplementary file 2 [file mmc2.docx]

**Appendix 2: Fundal Height and Birth Weight**

**Gestation Related Average Weight (GRAW) tool: Gestation Network**

<http://www.gestation.net/fetal_growth/graw/download_graw_chart.htm>

**Antenatal growth charts:** Gardosi J, Francis A. Customised Antenatal Growth Chart - GROW version 1, Year 2010 Gestation Network, [www.gestation.net](http://www.gestation.net) accessed 15 Nov 2015

***
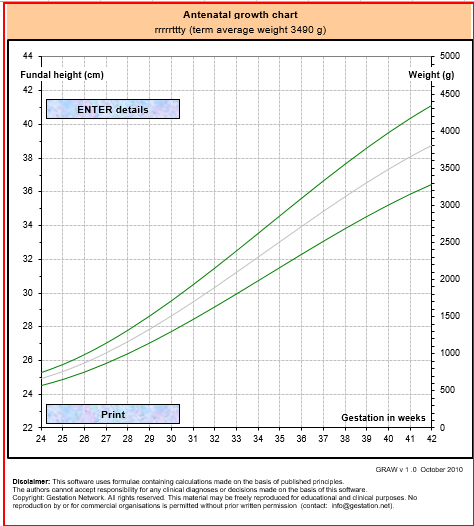
***
